# Temperature effects on fish production across a natural thermal gradient

**DOI:** 10.1111/gcb.13233

**Published:** 2016-03-03

**Authors:** Eoin J. O'Gorman, Ólafur P. Ólafsson, Benoît O. L. Demars, Nikolai Friberg, Guðni Guðbergsson, Elísabet R. Hannesdóttir, Michelle C. Jackson, Liselotte S. Johansson, Órla B. McLaughlin, Jón S. Ólafsson, Guy Woodward, Gísli M. Gíslason

**Affiliations:** ^1^ Department of Life SciencesImperial College LondonSilwood Park Campus, Buckhurst Road, AscotBerkshireSL5 7PYUK; ^2^ Institute of Life and Environmental SciencesUniversity of IcelandAskja, Sturlugata 7Reykjavík101Iceland; ^3^ The James Hutton InstituteAberdeenAB15 8QHUK; ^4^ Norwegian Institute for Water Research (NIVA)Gaustadalléen 21OsloN‐0349Norway; ^5^ Institute of Freshwater FisheriesKeldnaholtReykjavík112Iceland; ^6^ Centre for Invasion BiologyDepartment of Zoology and EntomologyUniversity of PretoriaHatfield 0026GautengSouth Africa; ^7^ Department of BioscienceAarhus UniversitySilkeborgDenmark; ^8^ Institut National de la Recherche Agronomique (INRA)UMR 1347 Agroécologie17 rue Sully ‐ BP 86510Dijon21065France

**Keywords:** natural experiment, Arctic, Hengill, freshwater, *Salmo trutta fario*, PIT tag, mark‐recapture, ecosystem services

## Abstract

Global warming is widely predicted to reduce the biomass production of top predators, or even result in species loss. Several exceptions to this expectation have been identified, however, and it is vital that we understand the underlying mechanisms if we are to improve our ability to predict future trends. Here, we used a natural warming experiment in Iceland and quantitative theoretical predictions to investigate the success of brown trout as top predators across a stream temperature gradient (4–25 °C). Brown trout are at the northern limit of their geographic distribution in this system, with ambient stream temperatures below their optimum for maximal growth, and above it in the warmest streams. A five‐month mark‐recapture study revealed that population abundance, biomass, growth rate, and production of trout all increased with stream temperature. We identified two mechanisms that contributed to these responses: (1) trout became more selective in their diet as stream temperature increased, feeding higher in the food web and increasing in trophic position; and (2) trophic transfer through the food web was more efficient in the warmer streams. We found little evidence to support a third potential mechanism: that external subsidies would play a more important role in the diet of trout with increasing stream temperature. Resource availability was also amplified through the trophic levels with warming, as predicted by metabolic theory in nutrient‐replete systems. These results highlight circumstances in which top predators can thrive in warmer environments and contribute to our knowledge of warming impacts on natural communities and ecosystem functioning.

## Introduction

Recent predictions suggest that Earth's surface will warm on average by at least 1.5 °C over the next century, with temperature increases of up to 9 °C at higher latitudes (IPCC 2013). These changes will have significant impacts on biological communities because temperature determines the metabolic demand of individual organisms (Brown *et al*., 2004). Warming is expected to disproportionately affect higher trophic levels (Petchey *et al*., 1999; Arim *et al*., 2007), with experimental evidence that metabolic rates rise more rapidly than ingestion rates in warmer environments, leading to energetic inefficiency and predator starvation (Rall *et al*., 2010; Vucic‐Pestic *et al*., 2011). Changes in top‐down control are likely to have major implications for community structure (Jochum *et al*., 2012; Shurin *et al*., 2012) and so the indirect effects of warming through the food web may be even greater than direct physiological effects.

Warming impacts are likely to be especially strong in freshwaters, whose relatively discrete ecosystem boundaries constrain the potential for species range shifts to track thermal optima, unlike many terrestrial and marine taxa (Perry *et al*., 2005; Chen *et al*., 2011). This is particularly true for organisms with an entirely freshwater life cycle which must either exploit thermal refugia, adapt to the warmer conditions, or perish (Cunjak, 1996; Kaeding, 1996; Ebersole *et al*., 2001; Hoffmann & Sgrò, 2011). As ectotherms, fish cannot regulate their body temperature and so warming will directly alter physiological functions such as thermal tolerance, growth, metabolism, food consumption, and reproductive success (Fry, 1971; Ficke *et al*., 2007; Pörtner & Farrell, 2008). If increases in metabolic demand are not matched by increasing food availability or strategies to maximize energy intake, populations are likely to decline or go extinct (McDonald *et al*., 1996).

Individual‐level responses to warming will be determined by their position within the thermal performance curve (Pörtner & Farrell, 2008), as well as the magnitude and rate of temperature change (Ficke *et al*., 2007). Populations inhabiting the lowest latitudes of their geographic range are likely to approach their critical temperature with warming, resulting in reduced physiological performance, declining abundance, or extinction (Almodóvar *et al*., 2012). Conversely, populations at the highest latitudes of their range may experience increased production with warming, particularly if resource production also increases (Blanchard *et al*., 2012).

**Figure 1:**
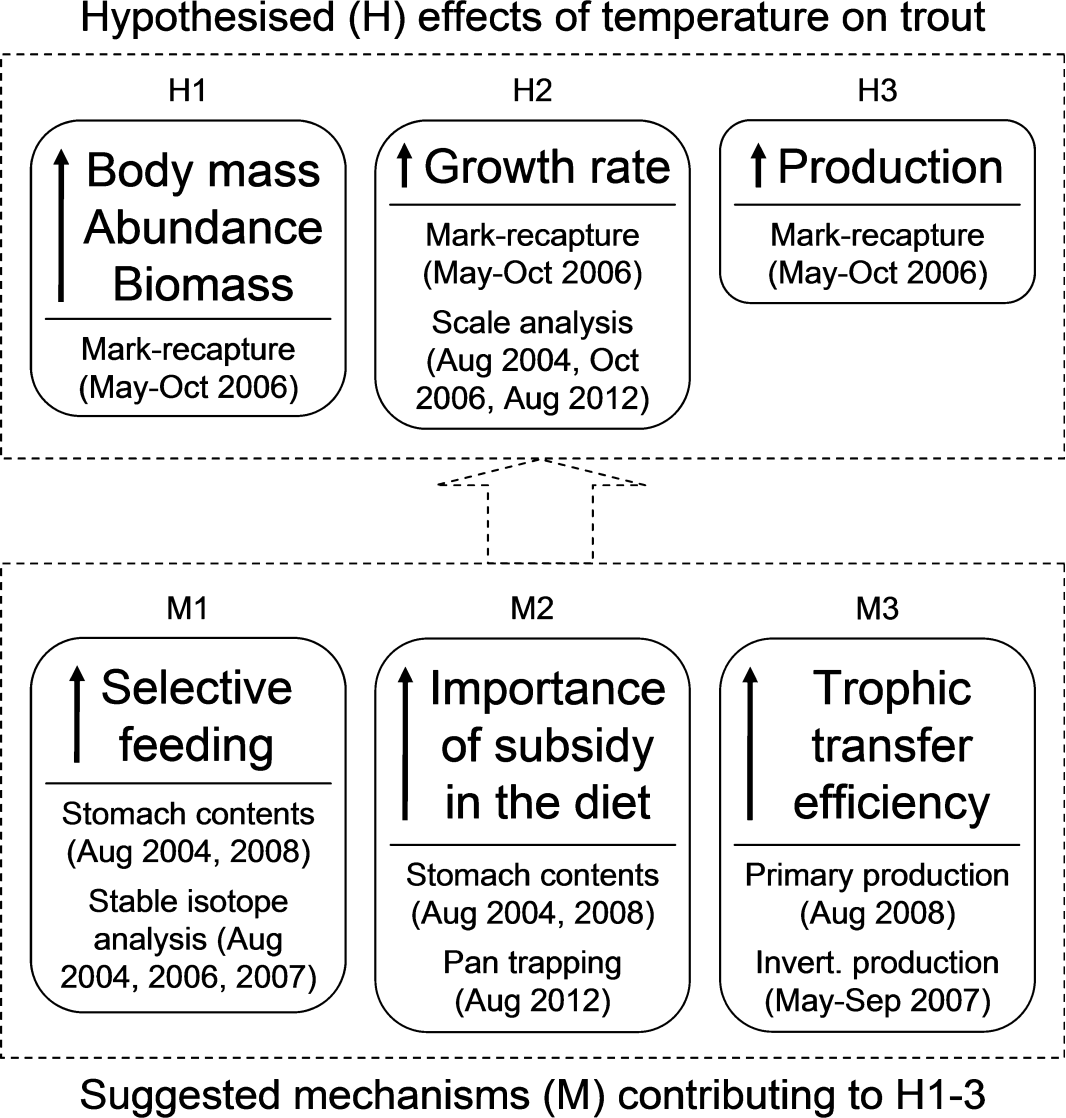
Overview of hypotheses and suggested mechanisms contributing to them, around which the paper is structured. Trout in our system are at the northern limit of their distribution, leading us to hypothesize that: (1) body mass, abundance and biomass; (2) growth rate; and (3) production will increase with temperature. We expect increases in: (1) feeding selectivity; (2) importance of external subsidy in the diet; and (3) trophic transfer efficiency at higher temperatures to facilitate our hypothesized effects on trout. Methodological approaches and timing of sampling are listed in smaller print beneath each metric.

We investigated the potential for apex predators to experience increased performance at high latitudes of their geographic range using brown trout, *Salmo trutta*, as a model apex predator across a natural stream temperature gradient in Iceland, which is the northern limit of the species (Elliott, 1994). Brown trout are ideal candidates as a model species because their ecophysiology is well‐known, they are widespread across the northern hemisphere, and they have the potential to influence ecosystem processes through top‐down effects in the food web (Elliott, 1994; Jonsson & Jonsson, 2009; Elliott & Elliott, 2010). Previous research in naturally heated streams indicates an increased growth rate, mean body mass, and population biomass of trout in warmer waters (Kaeding & Kaya, 1978; Woodward *et al*., 2010; O'Gorman *et al*., 2012). This may be due to adaptive strategies employed by trout to overcome the metabolic demands of the warmer environment, e.g. altered diet (Kaeding & Kaya, 1978; Woodward *et al*., 2010), or indirect effects of temperature on resource quantity or quality, e.g. both primary and secondary production increase with stream temperature in our study system (Gudmundsdottir *et al*., 2011; Hannesdóttir *et al*., 2013), while macroinvertebrate community composition also changes considerably (Friberg *et al*., 2009; Woodward *et al*., 2010). Thus, we hypothesize that mean body mass, population abundance and biomass, individual growth rates, and fish production will increase with stream temperature *via* three possible mechanisms: (1) more selective feeding behaviour; (2) greater importance of external subsidy in the diet; and (3) more efficient transfer of energy through the food web (see Fig. 1). We articulate the logic behind these proposed mechanisms below.

### Selective feeding

One potential mechanism to deal with the increased metabolic demand of a warmer environment is for consumers to actively choose higher quality resources. This strategy is comparable to the need for smaller herbivores to consume the most nutritious resources because they are limited by high metabolism and digestive constraints (Hopcraft *et al*., 2010). Thus, when energy requirements are low, a reasonable strategy for a generalist predator may be to have a lower search rate and consume a broad selection of mixed quality, but readily encountered prey. In warmer environments, however, it may be more beneficial to select for the most energetically valuable resources to lower the intake of resource biomass and overcome ingestion inefficiency (Rall *et al*., 2010; Vucic‐Pestic *et al*., 2011).

Warming is also likely to alter the composition and abundance of food resources available to higher trophic level consumers. Aquatic micro‐ and mesocosm experiments suggest an increase in the biomass of lower trophic level organisms with warming (Petchey *et al*., 1999; Yvon‐Durocher *et al*., 2011), although this may be dependent on top‐down control (O'Gorman *et al*., 2012; Shurin *et al*., 2012). Changes in community composition are also likely, with loss of species beyond their thermal limits and invasions of warm‐adapted taxa *via* range expansion (Lejeusne *et al*., 2010; Somero, 2010; Walther, 2010). Since fewer species will be closer to their optimum, a reduced subset of the community is likely to dominate under warming, leading to reduced evenness (Walker *et al*., 2006; Friberg *et al*., 2009; Pelini *et al*., 2011). Thus, even if the same number of prey species are available to predators, they are likely to encounter the dominant prey resources more regularly due to their elevated abundance, resulting in a narrower dietary niche breadth.

### External subsidy

Allochthonous inputs play a major role in freshwater ecosystems (Nakano & Murakami, 2001; Kawaguchi *et al*., 2003) and could potentially subsidise shortfalls in the energetic demand of aquatic consumers under warming (Cloe & Garman, 1996). For example, adult flies are a major dietary component of many freshwater fishes (Elliott, 1973; Cada *et al*., 1987; Fochetti *et al*., 2003; Kawaguchi *et al*., 2003) and altered larval emergence under warming may affect the timing, amount, and quality of this subsidy. For example, earlier emergence in warmer environments (Hogg & Williams, 1996; Greig *et al*., 2012) may lead to more generations within one year (Hannesdóttir *et al*., 2013) and thus a greater biomass of available subsidy. Alternatively, an increase in mean body size or the proportion of predatory flies with warming (Wesner, 2012; Jonsson *et al*., 2015) may provide a more energetically valuable resource (*per capita*) to drift‐feeding fish. These impacts may occur on true terrestrial subsidies due to warmer soil temperatures, or on the terrestrial adult phase of returning aquatic insects due to increased water temperatures. Greater input of falling arthropods as a result of warming in the terrestrial environment, e.g. through higher activity levels (Dell *et al*., 2011), could provide an additional subsidy which may help to alleviate the pressure of top‐down control on aquatic ecosystems (Nakano *et al*., 1999). Thus, greater availability of subsidy and the increasing inability to meet energy demands from in‐stream production may place greater importance on terrestrial invertebrates in the diet of drift feeding fish in warmer environments. While we predict that external subsidies will be more important in the diet of trout as stream temperature increases, we do not anticipate an increased biomass of subsidy to the warmer streams because air is not heated in the same way as the streams in our system and so this subsidy is likely to disperse evenly across the landscape.

### Trophic transfer efficiency

Biomass production at higher trophic levels is limited by the efficiency at which energy is transferred through the food web, with only about 10% of energy resource production being converted into consumer production (Pauly & Christensen, 1995; Ware, 2000; but see Slobodkin, 2001). Energy losses occur both within a trophic level (e.g. due to respiration and heat production) and between trophic levels (e.g. digestion efficiency and the energy required to encounter, capture, and handle a resource; Brown *et al*., 2004). A predator is likely to expend more energy searching for and handling its prey in a warmer environment, unless its preferred prey are sedentary (Vucic‐Pestic *et al*., 2011) or increase in abundance and thus availability with warming (Winfield *et al*., 2008). Trophic transfer efficiency is also related to the slope of the community size spectrum (Jennings & Mackinson, 2003), which is likely to change with increasing temperature (Yvon‐Durocher *et al*., 2011; O'Gorman *et al*., 2012). Shallower size spectra in the warmer streams of our study system are driven by fewer resources supporting more consumers (O'Gorman *et al*., 2012). Thus, we predict that energy is used more efficiently throughout the food web as stream temperature increases, mitigating the energetic demands of fish as apex predators in the warmer streams. We can make this prediction about trophic transfer efficiency quantitative with activation energies derived from the metabolic theory of ecology (Brown *et al*., 2004; Anderson‐Teixeira *et al*., 2008; Cross *et al*., 2015). We therefore expect the combination of all three drivers (selectivity, subsidy, and trophic transfer efficiency) to increase fish production at higher stream temperatures.

## Materials and methods

### Field site

All fieldwork was carried out in the Hengill geothermal valley of SW Iceland (N 64°03; W 21°18), which has numerous small tributaries that join the River Hengladalsá. Fourteen streams occur within 1.5 km of each other and span a temperature gradient of 4–25 °C (see Fig. S1; Friberg *et al*., 2009; Woodward *et al*., 2010; Demars *et al*., 2011; O'Gorman *et al*., 2012). Above‐ambient stream temperatures are due to groundwater that absorbs heat from the underlying rock rather than direct upwelling of geothermal water and gases (Arnason *et al*., 1969). Thus, while we cannot be certain that temperature is the only environmental factor leading to biotic differences between the streams, they are very similar in many other physical and chemical features (see Friberg *et al*., 2009; Adams *et al*., 2013 for detailed explorations). Trees are absent and shrubs are sparse in the region, so there is little or no particulate allochthonous input to the system; adult flies landing on the water surface are the major external subsidy (Friberg *et al*., 2009; Woodward *et al*., 2010). We carried out a number of studies in Hengill during the period 2004–2012 to assess how temperature affects the biomass, growth, and production of brown trout, the only fish species found in the system. Logistical and financial constraints meant that sampling these different components was often staggered over this period. We acknowledge that this requires some inferences across multiple years of data, but contend that this drawback is offset by studying temperature effects on trout populations under real world conditions. Sampling dates are summarized in Fig. 1 and mean stream temperatures for each study period are provided in Table 1.

**Table 1:** Stream codes and their corresponding daily mean temperatures during the sampling periods from the current study (note that 2008^trout^ refers to the mean for 27th July–16th August, while 2008^GPP^ are means during the 24‐h periods when PAR and GPP were measured from 6 to 16th August). Stream numbers correspond to those used in previous publications from Hengill (e.g. O'Gorman *et al*., 2012), with 16^upper^ and 16^lower^ corresponding to stretches of the River Hengladalsá that are upstream and downstream of a waterfall barrier, respectively. Note that we also refer to these river reaches as ‘streams’ throughout the paper for simplicity. See Fig. S1 for a photographic representation of all streams in the system

Stream	Temperature (°C)					
	2004	2006	2007	2008^trout^	2008^GPP^	2012
1	19.9	21.8	13.5	22.7	18.9	16.7
2	–	–	–	–	19.9	18.4
3	–	–	–	23.7	22.6	19.0
4	13.3	–	–	–	10.1	13.1
5	19.8	19.8	16.1	21.3	20.6	17.6
6	19.1	–	13.3	21.0	19.6	19.0
7	–	–	5.4	–	7.2	5.8
8	23.4	21.6	21.3	–	25.0	20.3
9	–	–	9.7	–	18.2	14.0
10	–	–	–	–	4.9	5.1
11	–	–	5.3	–	12.8	8.1
12	14.3	–	–	15.5	13.5	–
13	–	7.9	–	–	–	6.9
14	–	–	–	–	9.0	9.6
16^upper^	–	11.8	–	–	–	–
16^lower^	–	15.0	–	14.5	–	13.7

### Mark‐recapture study

We carried out a mark‐recapture study between May and October of 2006 to quantify body mass, abundance, biomass, growth rate and production of brown trout (testing H1, H2, and H3 in Fig. 1). Fish were captured by electrofishing and subsequently anesthetized with 2‐phenoxyethanol (roughly 1 ml per 3 l of stream water). Fork length and wet weight of every individual were measured before implanting Passive Integrated Transponders (PIT tags, supplied by Biomark Inc., Boise, ID, USA) in the abdominal cavity. The animals were allowed to recover briefly before being released back into the stream at the site where they were caught. A total of 394 trout (all ≥75 mm fork length) were tagged in this manner in six streams from 16–30th May 2006. Each PIT tag has a unique code, which is transmitted *via* a Biomark FS‐2001 field scanner, allowing each individual and its location to be recorded without physical disturbance by scanning the length of each stream every two weeks until 22nd October 2006, when the fork length and wet weight of the recaptured fish were measured once again. We determined a length**–**weight relationship for trout in the system from these data (see Fig. S2).

The population abundance (*N*) of trout in a stream was calculated using Chapman's estimator, a modification to the Lincoln‐Petersen mark‐recapture method to account for bias at small sample sizes (Chapman, 1951):

$$N=\left(\frac{\left(m+1\right)\left(c+1\right)}{r+1}-1\right)/a$$

where *m* is the number of fish marked on the first sampling occasion, *c* is the number of fish captured on the last sampling occasion, *r* is the number of marked fish that were recaptured, and *a* is the surface area that was fished. Population biomass (*B*) was calculated as $\bar{M}$ * *N*, where $\bar{M}$ is the mean body mass of trout in the stream during the study period. Growth rate (*G*) was calculated as (*M*_2_ – *M*_1_)/*t*, where *M*_1_ and *M*_2_ are the initial and final weight of the recaptured trout, respectively, and *t* is the duration of the mark‐recapture study. Note that we did not recapture any fish in the coldest stream, so we only have growth measurements for five streams. Trout production, *P*_*trout*_, was calculated as $\bar{G}$ * *B* * *t*, where $\bar{M}$ is the geometric mean growth rate of trout in each stream (after Elliott, 1994). The effects of stream temperature on the mean body mass, abundance, biomass, growth rate, and production of trout were analysed using linear regressions.

### Scale analysis

As a longer term measure of growth rate, we estimated the body mass of trout after one year of growth from 5 to 10 scales collected from each fish during electrofishing for trout in August 2004 (four streams), October 2006 (four streams), and in August 2012 (five streams). We focused only on the first year of growth to standardize observations due to different ages at the time of capture. The scales were cleaned with 5% potassium hydroxide, treated by ultrasound in a water bath for 30 s, placed on a drop of water on a glass slide, and sealed with a cover slide. High resolution photos were taken using a Nikon Digital Sight DSFi1 (Nikon UK Ltd, Kingston, Surrey, UK) connected to a Leitz Labovert FS microscope and examined for annuli, i.e. the theoretical boundary between two annual zones of growth. One annual zone consists of a band of narrow spaced circuli (rings on a fish scale) and a band of more widely spaced circuli, which indicate periods of slow (winter) and fast (summer) growth, respectively (Shearer, 1992). The distance from the scale centre to the newest ring on the first winter band corresponds to one year of growth. Relationships between scale radius and fish length were established for each stream (see Fig. S3) and used to calculate fish length after one year of growth. Fish mass was then estimated from the length‐weight relationship shown in Fig. S2. We used a generalized linear mixed model (GLMM) to analyse the effect of stream temperature (a fixed effect) on the geometric mean growth of trout after one year, with year of sampling included in the model as a random effect.

### Stomach content analysis

We quantified the selectivity of trout feeding in the system in August 2004 and 2008 (six streams in both years) to explore M1 and M2 in Fig. 1. Here, we compared the proportional representation of various prey items (by biomass) in the stomach contents of trout with the prevalence of the same taxa in the stream benthos. Fish were captured by electrofishing and euthanized, with the stomach dissected out and stored in 70% ethanol. The benthos of the corresponding streams were quantified using five Surber samples (25 × 20 cm quadrat, 200 *μ*m mesh size), also stored in 70% ethanol. Stomach contents and stream benthos samples were identified to the highest possible taxonomic level and linear body size dimensions (i.e. head width, body length, or shell width) were measured under 100× magnification. Body masses of all individuals were estimated from length**–**weight relationships (see O'Gorman *et al*., 2012 for details). The biomass of each species was calculated as mean body mass multiplied by abundance in either the stomach or the stream (although see the *Pan trapping study* below for terrestrial invertebrates).

Prey were pooled into six taxonomic groupings in line with previous stomach content explorations from the system (O'Gorman *et al*., 2012): snails (comprising only the freshwater snail, *Radix balthica*); blackfly larvae (Simuliidae); nonbiting midge larvae (Chironomidae); predatory aquatic dipteran larvae (Empididae, Ephydridae, Muscidae, and Tipulidae); miscellaneous aquatic taxa (including Plecoptera, Trichoptera, Acari, Heteroptera, and Oligochaeta); and terrestrial invertebrates (mainly adult Diptera). The selectivity of trout feeding on each group (*S*) was quantified as:

$$S=\frac{d_{i}/b_{i}}{\sum_{j=1}^{n}d_{j}/b_{j}},i=1,\ldots ,n,$$

where *d* and *b* are the proportional biomass of each prey group in the diet and in the stream benthos, respectively (Chesson, 1983). The effect of stream temperature and year of sampling on the selectivity of trout for each prey group was analysed using permutational multivariate analysis of variance (permanova), which can include both categorical and continuous variables (Anderson, 2001). We used a combination of Principle Coordinates Analysis (PCoA) and linear regression analyses to determine the effect of the same explanatory variables on the selectivity of trout for each of the six prey groups. The permanova and PCoA were based on a Euclidean resemblance matrix, which is appropriate in situations where a zero‐value (no selectivity in this case) is relevant to the hypothesis being tested (Clarke *et al*., 2006).

### Stable isotope analysis

We collected carbon and nitrogen (*δ* ^13^C and *δ* ^15^N) stable isotope data in 2004, 2006, and 2007 for trout and macroinvertebrates to assess the trophic position and dietary niche width of trout (exploring M1 in Fig. 1). Between 3 and 27 individual trout were collected from each stream, with dorsal muscle removed and subsequently frozen in the laboratory. Note that trout can undergo marked ontogenetic diet shifts (Grey, 2001) and the diets of small trout in the Hengill streams differ from those of their larger conspecifics (O'Gorman *et al*., 2012). As a result, only trout larger than 10 cm in fork length were used in this analysis and no individuals larger than 29 cm were found. Macroinvertebrates were collected using a hand net and kept cool until individual taxa could be separated back in the laboratory, placed in Petri dishes filled with tap water, and kept over night at 5 °C to allow gut evacuation before being frozen individually. All material was freeze‐dried and homogenized before it was analysed using a continuous flow isotope ratio mass spectrometer (Europe Hydra 20/20, PDZ Europe) coupled with an elemental analyser (see Friberg *et al*., 2009 for more details).

To determine the average trophic position of trout, the difference in the mean *δ* ^15^N signature of Simuliidae and trout was calculated from five streams in 2004, three streams in 2006, and five streams in 2007 (note, this was the maximum number of streams where both taxa were found). As primary consumers, Simuliidae represent a baseline signature against which the relative trophic position of the trout can be measured, accounting for the possibility of a shift in the *δ* ^15^N signature of the basal resources in each stream (after Post, 2002; see also Woodward *et al*., 2010). A fractionation of 3.4 *δ* ^15^N ‰ was assumed to represent a full trophic level difference between consumer and resource (Post, 2002). We also quantified the dietary niche width of trout in each stream and year (see Table S1 for sample sizes) using Bayesian standard ellipse areas (SEA_B_), a bivariate estimate of a population's core isotopic niche in *δ* ^13^C and *δ* ^15^N space, calculated using Markov Chain Monte Carlo simulation with 10^4^ iterations for each group (Jackson *et al*., 2011). Since SEA_B_ can be influenced by spatial and temporal differences in isotopic baselines (Jackson *et al*., 2011), we also calculated SEA_B_ for each macroinvertebrate community (see Table S1 for sample sizes). We used a GLMM to analyse the effect of stream temperature (a fixed effect) on the relative trophic position and dietary niche of trout, with year of sampling included in the model as a random effect. SEA estimates (and SEA_B_ in particular) are robust to variations in sample size (Jackson *et al*., 2011), but we also carried out linear regression analyses to confirm this.

### Pan trapping study

To explore M2 in Fig. 1, we carried out pan trapping in 14 streams in August 2012 to quantify the daily input of adult flies, the major subsidy to the system from the terrestrial environment and a component of the trout diet (O'Gorman *et al*., 2012). Pan traps consisted of white plastic trays measuring 33.5 × 21.5 × 10 cm, filled to a depth of 5 cm with stream water and a few drops of surfactant to reduce surface tension. Three pan traps were positioned on flat rocks protruding just above the surface of every stream in the Hengill system for a 24 h period on three consecutive days. The contents of the pan traps were stored in 70% ethanol for identification and counting back in the laboratory. All individuals were weighed after drying at 80 °C for 48 h in a drying oven. In addition to quantifying the total biomass of subsidy to each stream, *P*_*sub*_, we quantified the percentage biomass with terrestrial origin and the percentage originating from in‐stream production. The effect of stream temperature on *P*_*sub*_ was analysed using linear regression.

### Trophic transfer efficiency exploration

To assess trophic transfer efficiency through the food web (M3 in Fig. 1), our production estimates were all standardized to g C m^−2^ day^−1^ (note that we also present our estimates of body mass, biomass, and growth rate of trout in carbon units). We derived a conversion factor for photosynthetically active radiation (PAR; 1 mol photon m^−2^ day^−1^ = 6.13 g C m^−2^ day^−1^) by relating the Q/W ratio (2.5 × 10^21^ photon s^−1^ kJ^−1^ =4.15 × 10^−3^ mol photon kJ^−1^; Morel & Smith, 1974) with the reciprocal of the energy content of glucose expressed in carbon units (15.7 kJ g^−1^ glucose = 25.4 × 10^−3^ g C kJ^−1^; Southgate & Durnin, 1970). We used atomic weights to convert gross primary production (GPP; 32 g O_2_ = 12 g C) and conversions derived from the carbon element analysis that we performed prior to the stable isotope analysis for the higher trophic levels: *C*_*T*_ = 0.439 * *DW* and *C*_*I*_ = 0.452 * *DW*, where *C*_*T*_ and *C*_*I*_ are grams of carbon for trout and invertebrates, respectively, and *DW* is dry weight in grams. Invertebrate production, *P*_*inv*_, was previously reported (from six streams) for the period 30th September 2006 to 23rd August 2007 (Hannesdóttir *et al*., 2013). Here, we recalculated *P*_*inv*_ for 18th May to 23rd August 2007 to align it more closely with the time period for trout production. PAR and GPP were measured in 13 streams between 6–16th August 2008 (see Demars *et al*., 2011 for details). We present average PAR during this study period here because day to day differences in light availability had no impact on GPP (Demars *et al*., 2011). The effect of stream temperature on GPP and *P*_*inv*_ were analysed using linear regressions.

Trophic transfer efficiency was quantified as: (1) GPP/PAR; (2) *P*_*inv*_/GPP; (3) *P*_*trout*_/(*P*_*inv*_ + *P*_*sub*_). We have empirical estimates of transfer efficiency across all the trophic levels present for just three cold streams (IS7, 9, and 11, which do not contain fish) and three warm streams (IS1, 5, and 8, which do contain fish). We used a GLMM to analyse the effect of stream temperature (a fixed effect) on trophic transfer efficiency, with trophic level included in the model as a random effect.

### Quantitative predictions

We tested whether our empirical estimates of production and trophic transfer efficiency agreed with predictions based on the metabolic theory of ecology (Brown *et al*., 2004; Cross *et al*., 2015). Here, production, *P*, is proportional to body mass, *M*, and dependent on resource supply, [*R*], and temperature, *T*, with activation energy, *E*, and Boltzmann constant, *k *=* *8.62 × 10^−5^ eV °K^−1^:

$$P\propto \left[R\right]M^{0}e^{-\frac{E}{kT}}$$

The intrinsic activation energy of photosynthesis derived for terrestrial C_3_ plants (*E *≈* *0.32 eV; Allen *et al*., 2005) is inadequate for aquatic photosynthesizers due to their carbon‐concentrating mechanisms, which suppress photorespiration (Williams & Del Giorgio, 2005; Raven *et al*., 2012). The activation energy of whole stream GPP should therefore be similar to that of RUBISCO carboxylase activity, so we predict *E*_*GPP*_ ≈ 0.6 eV (Raven & Geider, 1988; Bernacchi *et al*., 2001; Galmés *et al*., 2015). We assume steady‐state conditions during the summer (Demars *et al*., 2011) and that previous observations of higher nutrient cycling and N_2_ fixation with increasing temperature will offset any nutrient limitation in the system (Rasmussen *et al*., 2011; Welter *et al*., 2015). Our prediction is likely to exceed the net value, however, as the model does not include the negative effect of grazing.

*P*_*inv*_ will be proportional to changes in resource supply (i.e. GPP) and its own temperature dependence, *E*_*inv*_ ≈ 0.6 eV, reflecting the average activation energy of enzymatic reactions (Elias *et al*., 2014) and a previous global synthesis (Golubkov, 2000). Thus, we predict that *P*_*inv*_ will increase with temperature with an activation energy of 1.2 eV (i.e. *E*_*GPP*_ + *E*_*inv*_; see Anderson‐Teixeira *et al*., 2008). Again, the predicted response to temperature will likely reflect the maximum because predation is not included in the model.

If *P*_*trout*_ relies solely on in‐stream invertebrate supply and its own temperature dependence, *E*_*trout*_ ≈ 0.6 eV (Golubkov, 2000; Elias *et al*., 2014), we would predict that *P*_*trout*_ will increase with temperature with an activation energy of 1.8 eV (i.e. *E*_*GPP*_ + *E*_*inv*_ + *E*_*trout*_). At the other extreme, if trout production relies solely on external subsidies (i.e. drift feeding as the sole feeding mechanism), then *P*_*trout*_ will not be related to in‐stream production. Assuming that *P*_*sub*_ is not related to stream temperature (because air temperatures are not heated in Hengill as for the streams), we would then expect *P*_*trout*_ to increase with temperature with an activation energy of 0.6 eV. Hence, the *P*_*trout*_ response to temperature is predicted to be 0.6 < *E*_*trout*_ < 1.8 eV, depending on the relative proportion of external subsidies to trout diet and assuming no resource limitation and no cannibalism.

The trophic transfer efficiency from PAR to GPP will have the same activation energy and slope as the temperature dependence of GPP because PAR is constant for all streams and due to our assumption of a nutrient‐replete system (Demars *et al*., 2011; Rasmussen *et al*., 2011; Welter *et al*., 2015), i.e. only the intercept of the relationship will change. All other trophic transfer efficiencies were hypothesized to have *E*_*TTE*_ ≈ 0.6 eV, based on the cellular activation energy of enzymatic reactions.

To test our predictions, we measured *E*_*GPP*_, *E*_*inv*_, *E*_*sub*_ and *E*_*trout*_ by extracting the intercept, *a*, and slope, *b*, of the observed empirical relationships between production and temperature according to the general regression equation log *P*_*i*_ = log *a*_*i*_ + *b*_*i*_ *T*_*i*_, where *i* corresponds to GPP, invertebrates, external subsidy, or trout. The proportional increase in temperature over 10 °C is $Q_{10}=e^{b_{i}\times 10}$, and activation energy (in eV) over the range 5–25 °C is *E*_*i*_ = *RT* ^2^ln(*Q*_10_/(*c* × 10)), where *R* is a gas constant (8.3 mol^−1^ K^−1^), *T* is the mean absolute temperature for the range over which *Q*_10_ was measured (288.15 K), and *c* is a constant to convert J mol^−1^ to eV (9.64 × 10^5^). The uncertainty in *E*_*i*_ was calculated as *E*_*i*_SE_*i*_/*b*_*i*_, where SE_*i*_ is the standard error of *b*_*i*_. We can assume that our results do not follow the metabolic theory of ecology if the predicted *E*_*i*_ falls outside the observed *E*_*i*_ ± 1.96 SE_*i*_, i.e. the 95% confidence intervals. The activation energy of the trophic transfer efficiencies were simply *E*_*TTE*_ = *E*_*j*_ – *E*_*i*_, where *i* and *j* are the lower and higher trophic levels, respectively. Note that we only quantify the efficiency of trout feeding solely on invertebrates (*P*_*trout*_/*P*_*inv*_) or subsidy (*P*_*trout*_/*P*_*sub*_). The uncertainties were estimated as $SE_{TTE}=E_{TTE}\sqrt{S{E_{j}}^{2}+S{E_{i}}^{2}}/\;\left(b_{j}-b_{i}\right)$. All analyses were carried out in r 3.1.0 using the ‘nlme’ package for GLMMs and the ‘siar’ package for stable isotope analysis (R Development Core Team 2014), with conditional *r* ^2^ values for GLMMs calculated after Nakagawa & Schielzeth (2013). Abundance, biomass, growth rate, and production data were ln‐transformed to meet the assumptions of linear regression and GLMM analyses.

## Results

**Figure 2:**
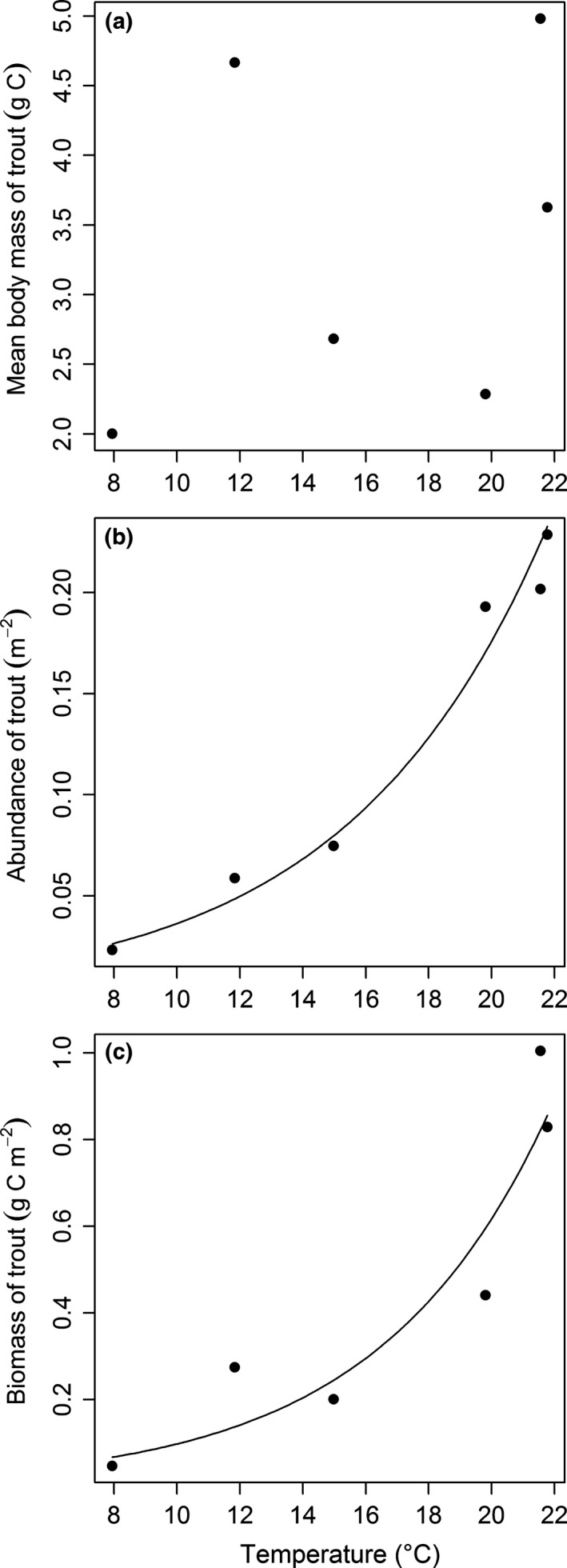
Relationships between stream temperature and: (a) mean body mass (Linear regression: *F*_1,4_ = 0.76, *P *=* *0.433); (b) population abundance (Linear regression: log *y *= log 0.0075 + 0.1578*x*,* F*_1,4_ = 194.4, *P *<* *0.001, *r* ^2^ = 0.97); (c) biomass (Linear regression: log *y *= log 0.0154 + 0.1844*x*,* F*_1,4_ = 28.02, *P *=* *0.006, *r* ^2^ = 0.84) of trout.

**Figure 3:**
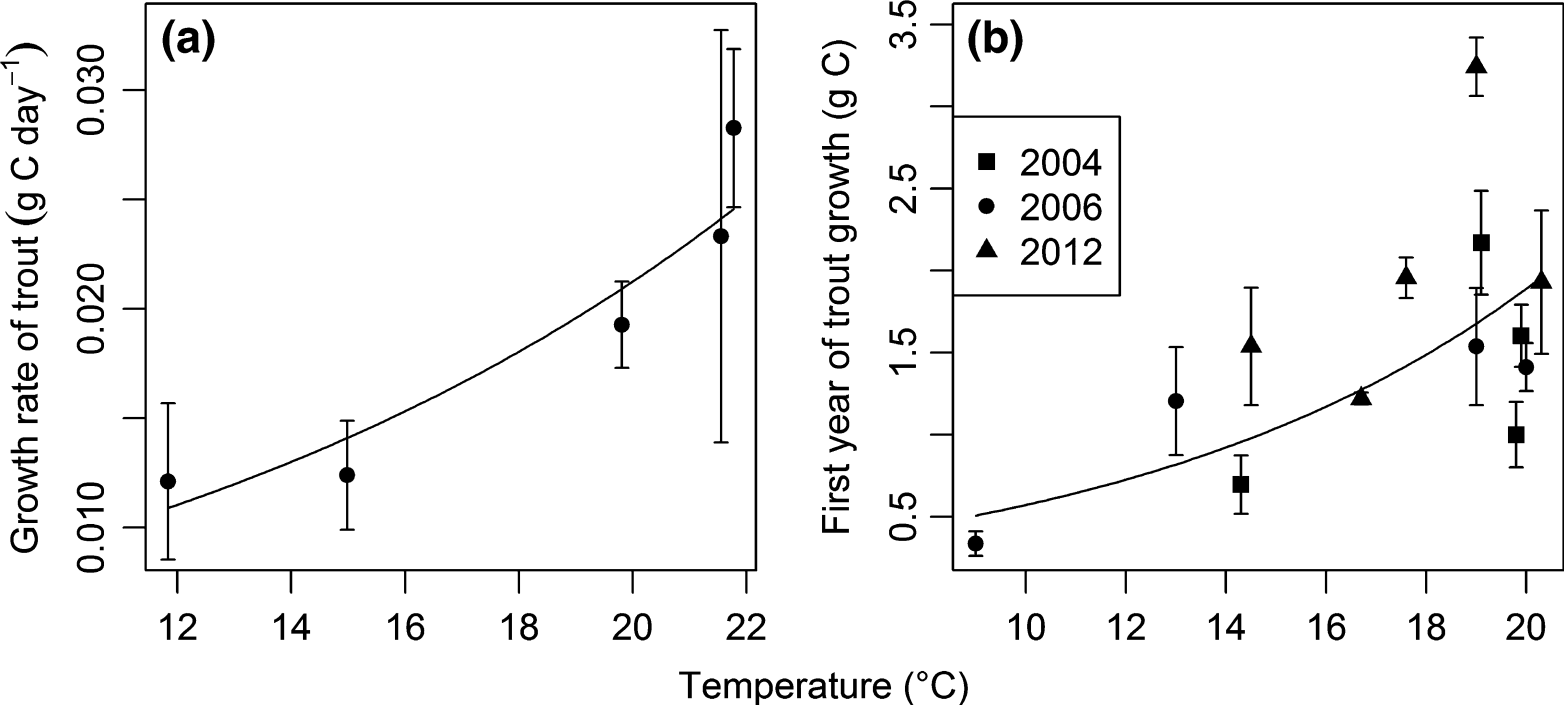
Relationships between stream temperature and: (a) geometric mean growth rate of trout in grams of carbon per day (Linear regression: log *y *= log 0.0041 + 0.0817*x*,* F*_1,4_ = 28.00, *P *=* *0.0132, *r* ^2^ = 0.87); (b) geometric mean grams of carbon after the first year of growth (GLMM: log *y *= log 0.1730 + 0.1195*x*,* t*_9_ = 3.817, *P *=* *0.004, *r* ^2^ = 0.63). Since growth rates are calculated from individual trout measurements, standard error bars are displayed for each stream.

**Figure 4:**
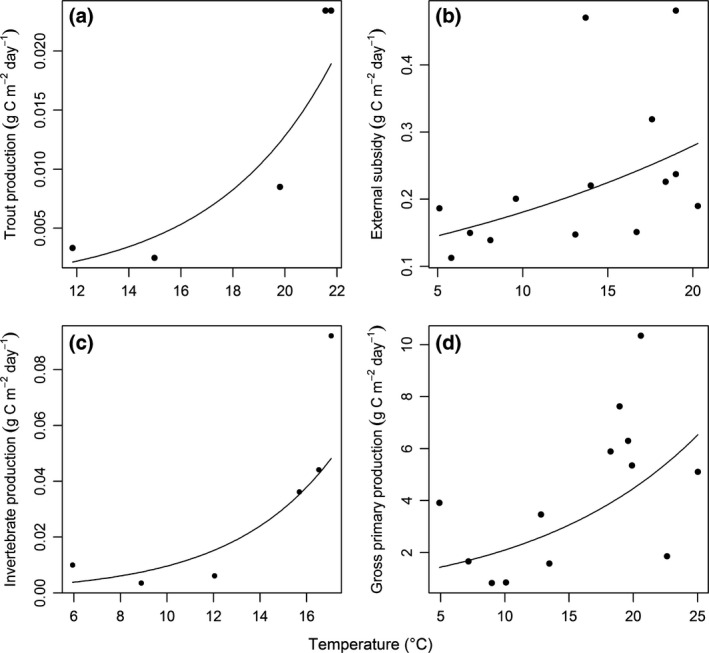
Relationships between stream temperature and: (a) trout production (Linear regression: log *y *= log 0.00016 + 0.2192*x*,* F*_1,3 _= 15.11, *P *=* *0.030, *r* ^2^ = 0.78); (b) subsidy of adult Diptera to the streams (Linear regression: log *y *= log 0.1171 + 0.0435*x*,* F*_1,12_ = 4.85, *P *=* *0.048, *r* ^2^ = 0.23); (c) benthic invertebrate production (Linear regression: log *y *= log 0.00098 + 0.2282*x*,* F*_1,4_ = 7.79, *P *=* *0.049, *r* ^2^ = 0.58); and (d) gross primary production (GPP) (Linear regression: log *y *= log 0.9857 + 0.0756*x*,* F*_1,11_ = 5.52, *P *=* *0.039, *r* ^2 ^= 0.27).

A total of 59 trout were recaptured of the 394 tagged in our study (see Table S2 for PIT tag scanning details at 10 different sampling points). Of the recaptured trout, 55 were located within 10 metres of the position where they were originally released, while just four had migrated to new streams in the system (see Table S2 for more details). There was no relationship between the mean body mass of trout and stream temperature (Linear regression: *F*_1,4_ = 0.76, *P *=* *0.433; Fig. 2a), but both the population abundance (Linear regression: *F*_1,4_ = 194.4, *P *<* *0.001; Fig. 2b) and biomass (Linear regression: *F*_1,4_ = 28.02, *P *=* *0.006; Fig. 2c) of trout increased with temperature in May 2006. Mean growth rate during the mark‐recapture study increased significantly as stream temperature increased (Linear regression: *F*_1,4_ = 28.00, *P *=* *0.0132; Fig. 3a). The body mass of trout after one year of growth also increased significantly with increasing stream temperature (GLMM: *t*_9_ = 3.817, *P *=* *0.004; Fig. 3b) as did production (Linear regression: *F*_1,3_ = 15.11, *P *=* *0.030; Fig. 4a).

**Figure 5:**
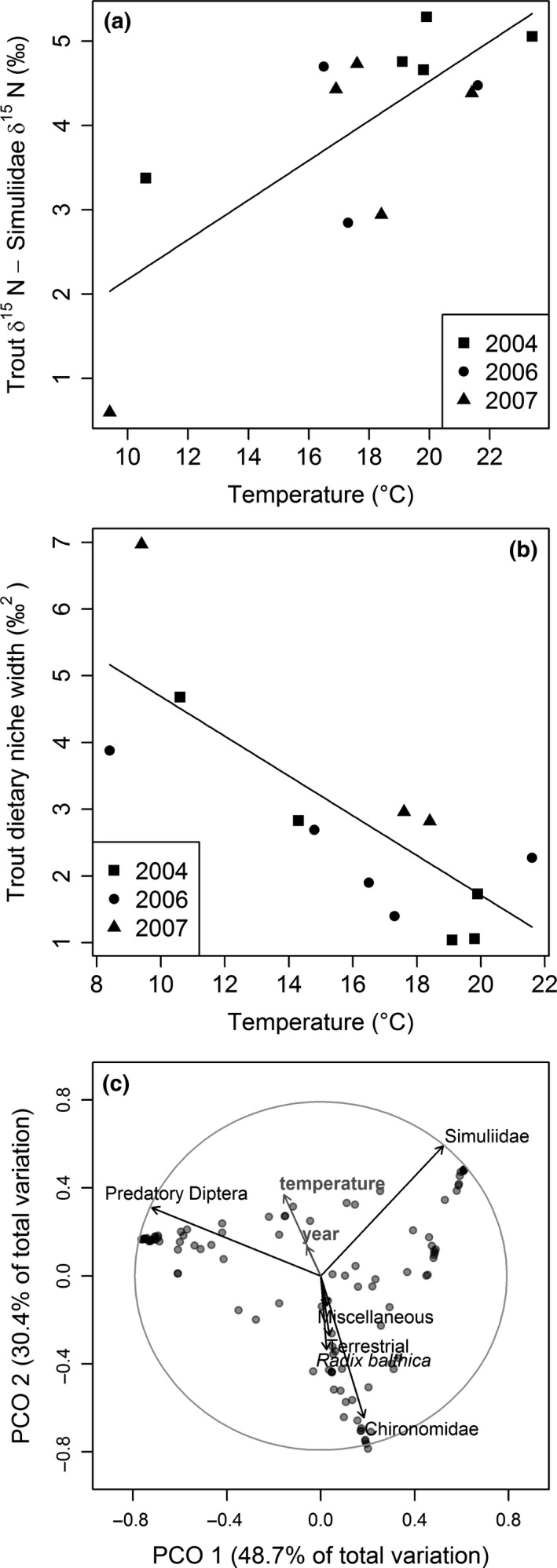
Relationships between stream temperature and the (a) average trophic position of trout relative to filter feeding Simuliidae (GLMM:* y *=* *0.2350*x* – 0.1742; *t*_9 _= 3.615, *P *=* *0.006; *r* ^2^ = 0.56); and (b) dietary niche width of trout (GLMM:* y *=* *−0.2979*x* + 7.6689; *t*_9_ = −5.326, *P *<* *0.001; *r* ^2^ = 0.77). (c) Euclidean ordination based on the resemblance matrix created from trout selectivity for six prey groups. The plot facilitates comparison of trout selectivity for the six prey groups (black arrows) with the predictor variables temperature and year (grey arrows) in multivariate space. Longer vectors indicate a stronger correlation. Darker symbols indicate one or more points overlaying each other.

Stable isotope analysis showed that the trophic position of trout increased (GLMM: *t*_9_ = 3.615, *P *=* *0.006; Fig. 5a) and dietary niche width decreased (GLMM: *t*_9_ = −5.326, *P *<* *0.001; Fig. 5b and Fig. S4) with stream temperature. The decline in dietary niche width signifies more selective feeding by trout in the warmer streams and occurred even though there was no significant change in the niche width of their macroinvertebrate prey (GLMM: *t*_7_ = 0.854, *P *=* *0.421; see Fig. S4). Note that there was no significant relationship between sample size and either trout (Linear regression: *F*_1,11_ = 2.53, *P *=* *0.14) or macroinvertebrate (Linear regression: *F*_1,9_ = 0.38, *P *=* *0.55) niche width. Stomach content analysis also revealed a main effect of temperature on the selectivity of trout feeding (permanova: MS = 4.431, pseudo‐*F *=* *11.37, *P *<* *0.001), with PCoA analysis indicating greater selectivity for aquatic predatory Diptera and Simuliidae and lower selectivity for all other prey groups as temperature increased (Fig. 5c). There was a significant interaction between temperature and year (permanova: MS = 1.162, pseudo‐*F *=* *2.98, *P *=* *0.036), with linear regression analysis indicating that this may be driven by the absence of a relationship between selectivity and stream temperature for Simuliidae and miscellaneous aquatic larvae in 2008 (see Fig. S5 and Table S3).

**Figure 6:**
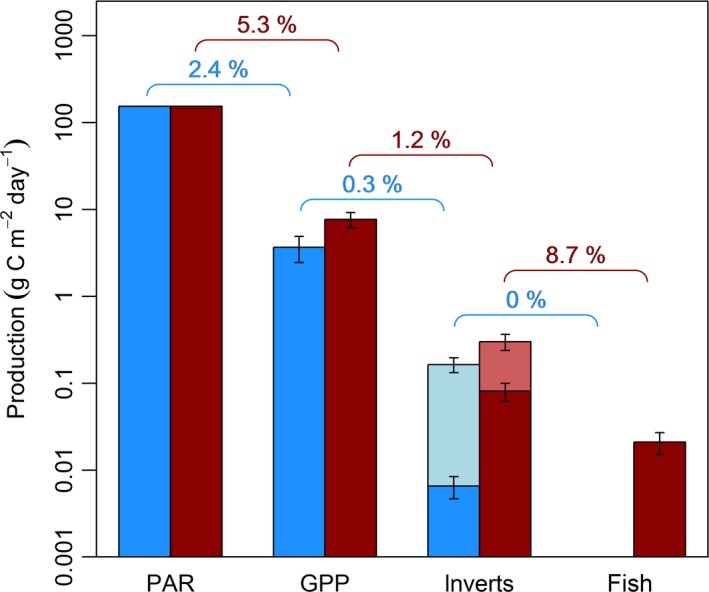
Mean trophic transfer efficiencies between each trophic level in the system for three cold (in blue) and three warm (in red) streams. Error bars represent the standard error around the mean for the three streams in each category. PAR is the same for every stream in the system, so no error bars are shown. Production estimates are expressed in g C m^−2^ day^−1^ at each trophic level for consistent comparison, with external subsidy of adult Diptera to the streams highlighted in pale blue and red. Mean invertebrate production in the cold and warm streams was 0.0066 and 0.0810 g C m^−2^ year^−1^, respectively; mean external subsidy in the cold and warm streams was 0.1637 and 0.3011 g C m^−2^ day^−1^, respectively.

The amount of external subsidy to the streams (Linear regression: *F*_1,12_ = 4.85, *P *=* *0.048; Fig. 4b), invertebrate production (Linear regression: *F*_1,4_ = 7.79, *P *=* *0.049; Fig. 4c), and GPP (Linear regression: *F*_1,11_ = 5.52, *P *=* *0.039; Fig. 4d) all increased with stream temperature. Note that 83 ± 5.8% of the external subsidy consisted of adult Diptera with a larval stage in terrestrial habitats: i.e., they were not derived from aquatic larvae. There was also a significant increase in trophic transfer efficiency with stream temperature (GLMM: *y *=* *0.0398*x* + 0.0088; *t*_14_ = 2.731, *P *=* *0.016, *r* ^2^ = 0.38; Fig. 6). Note that the *P*_*trout*_/(*P*_*inv*_ + *P*_*sub*_) trophic transfer efficiency here was 8.7%, while it would need to be 32.3% to sustain the same *P*_*trout*_ from *P*_*inv*_ alone.

The observed activation energies of GPP, *P*_*inv*_, and *P*_*trout*_ were all within the 95% confidence intervals of our theoretical predictions (Table 2). The activation energy of *P*_*sub*_ was greater than our prediction of zero, indicating a greater availability of external subsidy as stream temperature increased (Table 2). The change in trophic transfer efficiency with increasing temperature followed metabolic theory of ecology predictions for all trophic levels, although there was a large amount of uncertainty surrounding the observed activation energies (Table 2).

**Table 2:** Empirical estimates and theoretical predictions of the temperature dependence of production and trophic transfer efficiencies. Activation energies, *E*, are given in eV, along with the 95% confidence intervals (CI) of the empirically estimated value. Predictions that fall outside the 95% CI are highlighted in bold

Metric	Predicted *E* (eV)	Observed *E* (eV)	95% CI of observed *E* (eV)
GPP	0.6	0.54	0.08–1.00
*P*_*inv*_	1.2	1.63	0.46–2.80
*P*_*sub*_	**0**	0.31	0.03–0.59
*P*_*trout*_	0.6–1.8	1.57	0.76–2.37
GPP/PAR	0.6	0.54	0.08–1.00
*P*_*inv*_/GPP	0.6	1.09	−0.17–2.35
*P*_*trout*_/*P*_*inv*_	0.6	−0.06	−1.48–1.36
*P*_*trout*_/*P*_*sub*_	0.6	1.26	0.40–2.11

## Discussion

Warming is often predicted to have negative impacts on top predators (Petchey *et al*., 1999; Rosa & Seibel, 2008; Daufresne *et al*., 2009), but this study demonstrates that biomass, growth, and production of fish may increase with warming at high latitudes of their geographic range. These effects were driven by at least two mechanisms in the warmer streams of our study site: (1) fish selectively fed on highly abundant blackfly larvae and large predatory invertebrates as stream temperature increased, leading to a narrower dietary niche and a higher trophic position (Fig. 5); and (2) energy was converted into higher trophic level production more efficiently throughout the food web in the warmer environment (Fig. 6). Even though more adult Diptera landed on the surface of the warmer streams (Fig. 4b), there was no evidence for increased importance in the diet of trout, which actively selected against this external subsidy (Fig. 5c). Thus, through knowledge of environmental and biotic conditions and mechanisms to maximize energy intake, we can better predict the effects of warming on apex predators in natural systems.

### Thermal limits of the apex predator

The stream temperature gradient at Hengill falls within the critical temperature range for survival of fry, parr, and smoult of brown trout (approximately −0.8–30 °C) (Elliott & Elliott, 2010). The critical upper thermal limit for development of brown trout embryos is reported to be between 13–16 °C (Elliott, 1981; Jungwirth & Winkler, 1984; Ojanguren & Brana, 2003), however, suggesting that trout cannot reproduce in the warmest Hengill streams (see Kaya, 1977). Therefore, warm water trout populations are sustained either through use of colder waters for reproduction and eventual return of developed offspring, or recruitment of new individuals from coldwater populations (e.g. the main river stem). This is an important point when considering our results in the context of future climate change, which may remove the coldwater thermal refugia necessary for fish populations to be sustainable after warming beyond the critical temperature range of any part of their life cycle (e.g. Kaya, 1977; Meisner *et al*., 1988; Cunjak, 1996; Ebersole *et al*., 2001).

The optimum temperature for growth in brown trout fed on maximum rations of pelleted food in the laboratory is 11.6–19.1 °C (Ojanguren *et al*., 2001; Forseth *et al*., 2009; Elliott & Elliott, 2010). The coldest streams in the Hengill system fall below this range and so it is perhaps unsurprising that trout are rarely found in them, although the lower critical limit for growth in the species is 1.2 °C (Ojanguren *et al*., 2001; Elliott & Elliott, 2010), so they should still be able to survive and grow there (Table S2). Surprisingly, the three warmest streams in the system (19.8–21.8 °C) are above the documented optimum temperature for maximum growth and well above those studies that focus on invertebrate food only (13.1–14.1 °C; Elliott *et al*., 1995; Elliott & Elliott, 2010). This suggests that we may have expected to see a hump‐shaped response to temperature, with growth increasing to the optimum before dropping off at higher temperature (although see Kaeding & Kaya, 1978), rather than the exponential increases described (Fig. 3). The highly controlled laboratory studies that growth performance curves are typically based on may not reflect the actual thermal optima for growth in field conditions, e.g. due to local environmental conditions or diversity of resources available. Quality of the resource is also likely to be important and thus the optimum temperature for growth may change with diet (Kaeding & Kaya, 1978; Elliott & Elliott, 2010). It should be noted that the growth performance studies focusing on invertebrate food were carried out solely with amphipods (Elliott *et al*., 1995), which are not found in our system. Additionally, long‐term adaptation to warmer temperatures in the Hengill system (Arnason *et al*., 1969) may result in higher local thermal optima (Schulte *et al*., 2011; Huey *et al*., 2012). This highlights the potential for intraspecific variation and the context dependency of laboratory‐based thermal performance curves for application in real ecosystems. Naturally occurring thermal gradients, such as at Hengill, could thus also be useful as natural laboratories for assessing thermal performance of individual organisms under more realistic field conditions (O'Gorman *et al*., 2014).

### Selective feeding

Trout clearly changed their feeding behaviour as stream temperature increased by narrowing their diet and altering their selectivity (Fig. 5b, c). This may be partly explained by changes in the community composition of their prey, with the decreasing preference for Chironomidae vs. Simuliidae reflecting the switch in dominance of these two groups in the warmer streams (Friberg *et al*., 2009; Woodward *et al*., 2010). There was also clear evidence for changes in feeding behaviour of trout that were not driven by shifts in macroinvertebrate composition. There was a decline in selectivity for snails with increasing temperature, even though they account for the largest prey biomass in the warmest streams (O'Gorman *et al*., 2012). This may be a product of the lower caloric content of snails relative to the similarly abundant blackfly larvae (Cummins, 1967; Griffiths, 1977). Trout also exhibited a distinct preference for predatory dipteran larvae, despite their relative rarity in the benthos (Woodward *et al*., 2010), perhaps because their high caloric and protein diet acts as a richer food source than primary consumers (Cummins & Klug, 1979). This increasing selectivity for prey with a high energetic value in warmer waters may allow the trout to meet their greater metabolic requirements.

Mean trout body mass was similar across sites along the temperature gradient during the mark‐recapture study (May–Oct 2006), contradicting our first hypothesis and previous evidence from the Hengill system (Woodward *et al*., 2010; O'Gorman *et al*., 2012), although this might simply reflect Type II errors associated with a small sample size. The previously observed increases in trout body mass with temperature occurred in 2004 and 2008 (Woodward *et al*., 2010; O'Gorman *et al*., 2012), matching the timing of our dietary analyses (see Fig. 1). These increases in body size in the warmer streams may have affected the diet of the trout, with a shift towards larger prey to meet their optimal foraging requirements (Brose *et al*., 2008; Petchey *et al*., 2008). This may be a contributing factor in the greater selectivity for predatory Diptera, which are among the largest macroinvertebrates in the system, and blackfly larvae, whose body mass increases with temperature (O'Gorman *et al*., 2012). Note that this still did not result in greater selectivity for snails, which have previously been shown to increase in prevalence in the diet of larger trout (Steingrímsson & Gíslason, 2002).

### External subsidy

Adult flies landing on the surface of rivers and streams are a major component of salmonid diets (Elliott, 1973; Cada *et al*., 1987; Fochetti *et al*., 2003). In contrast with our expectations, trout actively selected against this external subsidy as stream temperature increased (Fig. 5c). This is particularly surprising given that we observed a higher external subsidy to the warmer streams (Fig. 4b), contrary with our quantitative prediction (Table 2). While we can predict a higher rate and biomass of emerging Diptera in warmer environments (Hannesdóttir *et al*., 2013), we might expect them to disperse evenly across the Hengill landscape because there is no gradient in air temperatures as there is for the streams. Several studies have indicated that adult Diptera show natal site fidelity when ovipositing (Rothfels, 1981; Martin *et al*., 2002; Hilker & Meiners, 2002; Krosch *et al*., 2011; but see Hunter & Jain, 2000), which would increase the likelihood of them remaining close to the location from which they emerged. This may be true for aquatic larvae emerging from the streams or terrestrial larvae emerging from soils, which also exhibit higher temperatures near the warm streams in Hengill. Additionally, some adult Diptera are known to aggregate around warmer waters for foraging, including two major components of our pan trapping study: Ephydridae, which feed on cyanobacteria; and the predatory Dolichopodidae (Brock *et al*., 1969).

External subsidies still play a role in the system, however, because in‐stream production alone is unlikely to sustain trout populations (Elliott, 1994). Our gut content analysis revealed that trout fed on terrestrial invertebrates in every stream studied (see also O'Gorman *et al*., 2012). Trophic transfer efficiency from the combined production of in‐stream invertebrates and external subsidy to trout was also close to the value of 10% reported for many ecosystems (Pauly & Christensen, 1995; Ware, 2000), but if trout relied solely on in‐stream production, this efficiency would have to rise to over 30% to sustain the same level of fish production (Fig. 6). The large amount of subsidy with a terrestrial origin (approximately 83%) confirms that this additional food source for trout is largely independent of in‐stream production, which may also help to alleviate the higher metabolic demands of the warmer environment. Future work should look at other terrestrial subsidies, such as the drift of ground‐dwelling terrestrial arthropods (e.g. Coleoptera, Hymenoptera), which also form part of the diet of trout (Elliott, 1973; Cada *et al*., 1987). Given the increased activity of terrestrial invertebrates in warmer environments (Dell *et al*., 2011), their contribution to the drift is likely to be greater with warming, creating an additional external subsidy to support drift‐feeding fish. In reality, any warming‐induced subsidies in the current study will be conservative estimates as the catchment experiences only localized terrestrial warming, whereas under a warmer climate this would span the entire catchment, thus potentially amplifying the effects even further.

### Trophic transfer efficiency

The activation energy of our empirical trophic transfer estimates conform with our predictions based on the metabolic theory of ecology (Table 2), highlighting the merit in making *a priori* quantitative predictions. This provides a deterministic approach against which expected effect size can be compared to observed data and is more objective than simply applying *a posteriori* explanations to the empirical patterns. Nevertheless, our knowledge of the study system helps to understand the underlying processes. Nitrogen is the main limiting nutrient in the Hengill streams (Friberg *et al*., 2009), but faster recycling of NH_4_ and NO_3_ (Demars *et al*., 2011) and a higher N_2_ fixation rate (Welter *et al*., 2015) facilitate a more efficient conversion of available sunlight to gross primary production in the warmer streams (Fig. 6). The standing stock of biofilm, the major basal resource in the food web, is relatively constant across the stream temperature gradient at Hengill (Friberg *et al*., 2009; Gudmundsdottir *et al*., 2011), even though there is a more rapid accumulation as temperature increases (Welter *et al*., 2015). This is most likely due to more intensive grazing pressure in the warmer streams (O'Gorman *et al*., 2012), illustrating how rapid replenishment of resources can meet the higher metabolic demand of heterotrophs in the warmer environment.

Primary production is in turn converted into secondary production more efficiently as temperature increases (Fig. 6), most likely due to the shift in functional feeding modes as the dominant macroinvertebrates switch from Chironomidae in the cold streams to Simuliidae and freshwater snails in the warm streams (Friberg *et al*., 2009; Woodward *et al*., 2010). Chironomidae selectively gather fine particles from the biofilm, whereas the freshwater snail is a highly efficient scraper, rasping biofilm from benthic substrates as it moves (Cummins & Klug, 1979). Simuliidae are largely sedentary organisms, which filter feed small organic particles from the water column (Wallace & Merritt, 1980). They have one of the highest growth conversion efficiencies among freshwater macroinvertebrates and are thus an important energy source for secondary consumers (Cummins & Klug, 1979; McCullough *et al*., 1979), who are also likely to expend less energy feeding on these sedentary organisms (Vucic‐Pestic *et al*., 2011). The combination of more efficient feeding by primary consumers on basal resources and the surprising increase in external subsidy to the warmer streams (Fig. 4b) may be a critical factor in sustaining trout populations relative to the colder (and less productive) environments (Fig. 6).

### A word of caution

It took us many years to collect all the data presented in this study, thus while each of our hypotheses is tested from samples collected at the same time, the full dataset that explores the underlying mechanisms was unavoidably drawn from different years (see Fig. 1). Patterns in trout diet, trophic group biomass, size spectra, and food web structure are consistent between years for the system (O'Gorman *et al*., 2012) and our multiple measures of trout growth rate further support this consistency (Fig. 3). Nevertheless, we cannot be certain that the temperature dependence of trout production was maintained throughout the sampling period of the underlying mechanisms and the trophic transfer efficiencies we report represent the best approximations we could achieve from the mix of sites and sampling times. Finally, trout were only present in six streams, which inevitably constrained the maximum sample size and strength of inferences we can draw for some of our analyses (see Figs 2 and 4a). We contend that these constraints are offset, however, by the unique advantages of being able to study the effects of temperature on trout as members of the wider ecosystem in a natural setting.

## Conclusion

Warming is widely predicted to cause reductions in mean food chain length (Petchey *et al*., 1999; Arim *et al*., 2007) and a shift towards smaller organisms (Daufresne *et al*., 2009; Gardner *et al*., 2011; Forster *et al*., 2012). This study demonstrates, however, that warming can enhance populations of apex predators when ambient temperatures are suboptimal for overall physiological performance. This highlights the need to identify the thermal limits and adaptive capacity of organisms to improve our predictive abilities of warming impacts on natural systems (Schulte *et al*., 2011; Huey *et al*., 2012). Knowledge of mechanisms that enable organisms to extract more energy from the environment are also critical, whether they are autecological (e.g. the dietary shifts shown here) or synecological (e.g. allochthonous subsidies, or more efficient transfer of energy through the system) in nature. This may allow higher trophic level organisms to meet the increased metabolic demands of living in a warmer environment and even thrive on the associated productivity. Global change is, of course, a multifaceted issue, with multiple stressors potentially combining to alter ecosystems in unexpected ways (Jackson *et al*., 2016). Thus, future research should aim to quantify synergistic or antagonistic effects of other stressors on warming to improve predictability even further.

## Supporting information


**Figure S1.** Map of the Hengill geothermal valley.
**Figure S2.** Length‐weight relationship for brown trout.
**Figure S3.** Scale radius to fish length relationships.
**Figure S4.** Dietary niche width of trout and invertebrates.
**Figure S5.** Selectivity in the feeding of trout on common prey groups.
**Table S1.** Sample sizes for estimating dietary niche width of trout and invertebrates
**Table S2.** Details of sampling occasions during the trout mark‐recapture study
**Table S3.** Linear regression statistics for selectivity of trout feeding
