# Supplementary material for: Temperature effects on fish production across a natural thermal gradient
Source: Glob Chang Biol. 2016 Mar 3;22(9):3206–20. doi: 10.1111/gcb.13233 (PMC4991275; doi:10.1111/gcb.13233)

Supporting Information: Temperature effects on fish production across a natural thermal gradient

Eoin J. O’Gorman^1,*,†^, Ólafur Patrick Ólafsson^2,†^, Benoît O.L. Demars^3^, Nikolai Friberg^4^, Guðni Guðbergsson^5^, Elísabet R. Hannesdóttir^2,5^, Michelle C. Jackson^6^, Liselotte S. Johansson^7^ , Órla B. McLaughlin^1,8^, Jón S. Ólafsson^5^, Guy Woodward^1^, and Gísli Már Gíslason^2,*^

*^1^ Department of Life Sciences, Imperial College London, Silwood Park Campus, Buckhurst Road, Ascot, Berkshire SL5 7PY, UK.*

*^2^ Institute of Life and Environmental Sciences, University of Iceland, Askja, Sturlugata 7, 101 Reykjavík, Iceland.*

*^3^ The James Hutton Institute, Aberdeen, AB15 8QH, Scotland, UK.*

*^4^ Norwegian Institute for Water Research (NIVA), Gaustadalléen 21, N-0349* *Oslo, Norway.*

*^5^ Institute of Freshwater Fisheries, Keldnaholt, 112 Reykjavík, Iceland.*

*^6^ Centre for Invasion Biology, Department of Zoology and Entomology, University of Pretoria, Hatfield 0026, Gauteng, South Africa.*

*^7^ Department of Bioscience, Aarhus University, Silkeborg, Denmark.*

*^8^ Institut National de la Recherche Agronomique (INRA), UMR 1347 Agroécologie, 17 rue Sully - BP 86510, 21065 Dijon, France.*

*** Corresponding authors:** Eoin O'Gorman; Gísli Már Gíslason

^†^ These authors contributed equally to this work

**E-mail:** e.ogorman@imperial.ac.uk; gmg@hi.is

**Table S1.** Sample sizes for estimating dietary niche width of trout and invertebrates in three separate years (2004, 2006, and 2007). Stream numbers correspond to those shown in Table 1 and Fig. S1. Number of trout and invertebrate individuals sampled for carbon and nitrogen (δ^13^C and δ^15^N) stable isotope signatures are provided, along with Bayesian standard ellipse areas (SEA_B_).

| **Year** | **Stream** | **Trout individuals** | **Invertebrate individuals** | **Trout SEA_B_** | **Invertebrate SEA_B_** |
| --- | --- | --- | --- | --- | --- |
| 2004 | 1 | 15 | 6 | 1.73 | 9.05 |
| 2004 | 5 | 16 | 7 | 1.06 | 2.57 |
| 2004 | 6 | 13 | 15 | 1.04 | 6.95 |
| 2004 | 12 | 27 | 13 | 2.83 | 3.61 |
| 2004 | 14 | 8 | 5 | 4.68 | 2.78 |
| 2006 | 5 | 7 | 5 | 1.40 | 3.29 |
| 2006 | 6 | 15 | 11 | 1.90 | 8.58 |
| 2006 | 8 | 5 | 6 | 2.27 | 4.52 |
| 2006 | 12 | 10 | - | 2.69 | - |
| 2006 | 14 | 6 | - | 3.88 | - |
| 2007 | 1 | 13 | 9 | 2.82 | 18.53 |
| 2007 | 5 | 4 | 6 | 2.96 | 5.78 |
| 2007 | 14 | 3 | 13 | 6.97 | 6.98 |

**Table S2.** Details of PIT tags that were picked up by the scanner on each of ten separate sampling occasions during the trout mark-recapture study. Sampling dates are given, along with stream ID and temperature (°C). The mean temperature of each stream (Temp) was estimated from spot measurements taken in 10 m intervals along the length of each stream. Standard errors (Temp SE) are also presented to illustrate the small amount of longitudinal variation in stream temperature. The total number of tagged trout that were located on each sampling occasion is given in bold. Of the 59 trout recaptured at the end of the study, 55 were within 10 metres of the location where they were originally released. One trout moved from the upstream reach of the river to 1,500 m downstream (past the waterfall barrier); one trout moved 135 m from the upstream reach of the river to stream 13; one trout moved 720 m from stream 5 to stream 1; and one trout moved 880 m from stream 1 to stream 5.

| **Stream** | **Temp** | **Temp SE** | **Tagged** | **16^th^ Jun** | **30^th^ Jun** | **14^th^ Jul** | **28^th^ Jul** | **12^th^ Aug** | **26^th^ Aug** | **9^th^ Sep** | **23^rd^ Sep** | **7^th^ Oct** | **22^nd^ Oct** |
| --- | --- | --- | --- | --- | --- | --- | --- | --- | --- | --- | --- | --- | --- |
| 13 | 7.9 | 0.02 | 2 | 2 | 1 | 0 | 0 | 0 | 0 | 0 | 0 | 0 | 0 |
| 16^upper^ | 11.8 | 0.52 | 35 | 27 | 21 | 19 | 13 | 9 | 7 | 6 | 6 | 6 | 6 |
| 16^lower^ | 15.0 | 0.46 | 135 | 124 | 77 | 61 | 39 | 17 | 10 | 5 | 4 | 4 | 3 |
| 5 | 19.8 | 0.05 | 108 | 107 | 91 | 68 | 46 | 33 | 28 | 26 | 23 | 23 | 21 |
| 8 | 21.6 | 0.11 | 19 | 19 | 14 | 12 | 8 | 5 | 4 | 3 | 2 | 2 | 2 |
| 1 | 21.8 | 0.06 | 95 | 85 | 70 | 49 | 42 | 30 | 27 | 27 | 27 | 27 | 27 |
|  |  |  | **394** | **364** | **274** | **209** | **148** | **94** | **76** | **67** | **62** | **62** | **59** |

**Table S3. Linear regression statistics for selectivity of trout feeding on six prey groups: the freshwater snail (*Radix balthica*), blackfly larvae (Simuliidae), midge larvae (Chironomidae), predatory dipteran larvae, miscellaneous aquatic prey, and terrestrial subsidy. Non-significant terms were removed from each model if AIC comparison revealed a smaller AIC value for the simpler model. See Fig. S5 for a visualisation of the data.**

|  |  | **Temperature** | | | **Year** | | | **Temperature : Year** | | |
| --- | --- | --- | --- | --- | --- | --- | --- | --- | --- | --- |
| **Prey group** | **Intercept** | **Slope** | ***t*** | ***p*** | **Slope** | ***t*** | ***p*** | **Slope** | ***t*** | ***p*** |
| *Radix balthica* | 0.5467 | -0.0246 | -3.687 | <0.001 |  |  |  |  |  |  |
| Simuliidae | -0.5800 | 0.0501 | 3.072 | 0.003 | 1.1281 | 2.784 | 0.006 | 0.0611 | -2.765 | 0.007 |
| Chironomidae | 0.6826 | -0.0254 | -3.035 | 0.003 | -0.0746 | -1.433 | 0.155 |  |  |  |
| Predatory Diptera | -0.6001 | 0.0510 | 4.538 | <0.001 |  |  |  |  |  |  |
| Miscellaneous | 0.4071 | -0.0197 | -3.194 | 0.002 | -0.3701 | -2.415 | 0.017 | 0.0180 | 2.160 | 0.033 |
| Terrestrial | 0.1405 | -0.0066 | -3.145 | 0.002 |  |  |  |  |  |  |

**Fig. S1.** Map of the Hengill geothermal valley with stream locations from the trout mark-recapture study indicated with solid black arrows. All other streams in the system are indicated by the dashed arrows. Stream labels correspond to those listed in Table S2. The position of a large waterfall barrier separating IS16 upper and lower (IS16 U and IS16L) is also shown on the map.

**
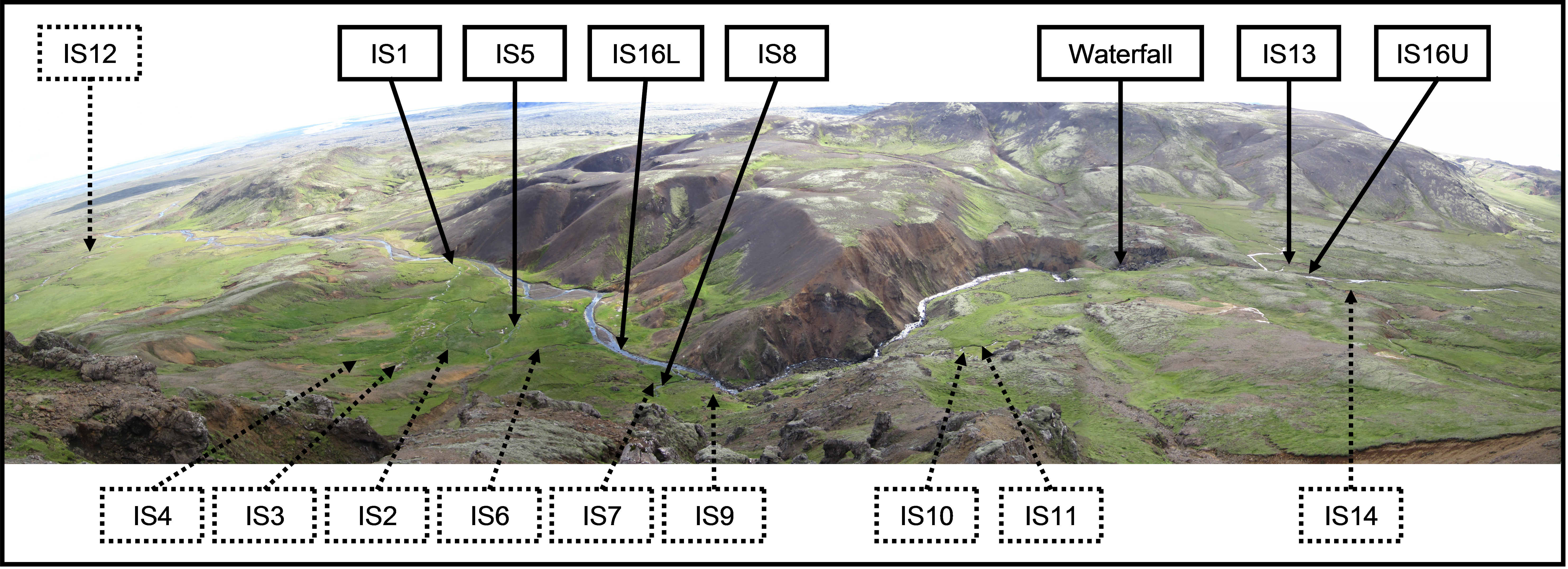
**

**Fig. S2.** Length-weight relationship for brown trout, *Salmo trutta*, in the Hengill system. Fork length (mm) and wet weight (g) were measured for 458 individuals during the period 16^th^ May to 22^nd^ October 2006. Linear regression: *y* = 3.02*x* – 5.000, *F*_1,456_ = 2.76×10^4^, *p* < 0.001, *r*^2^ = 0.98. Darker symbols indicate one or more points overlaying each other.


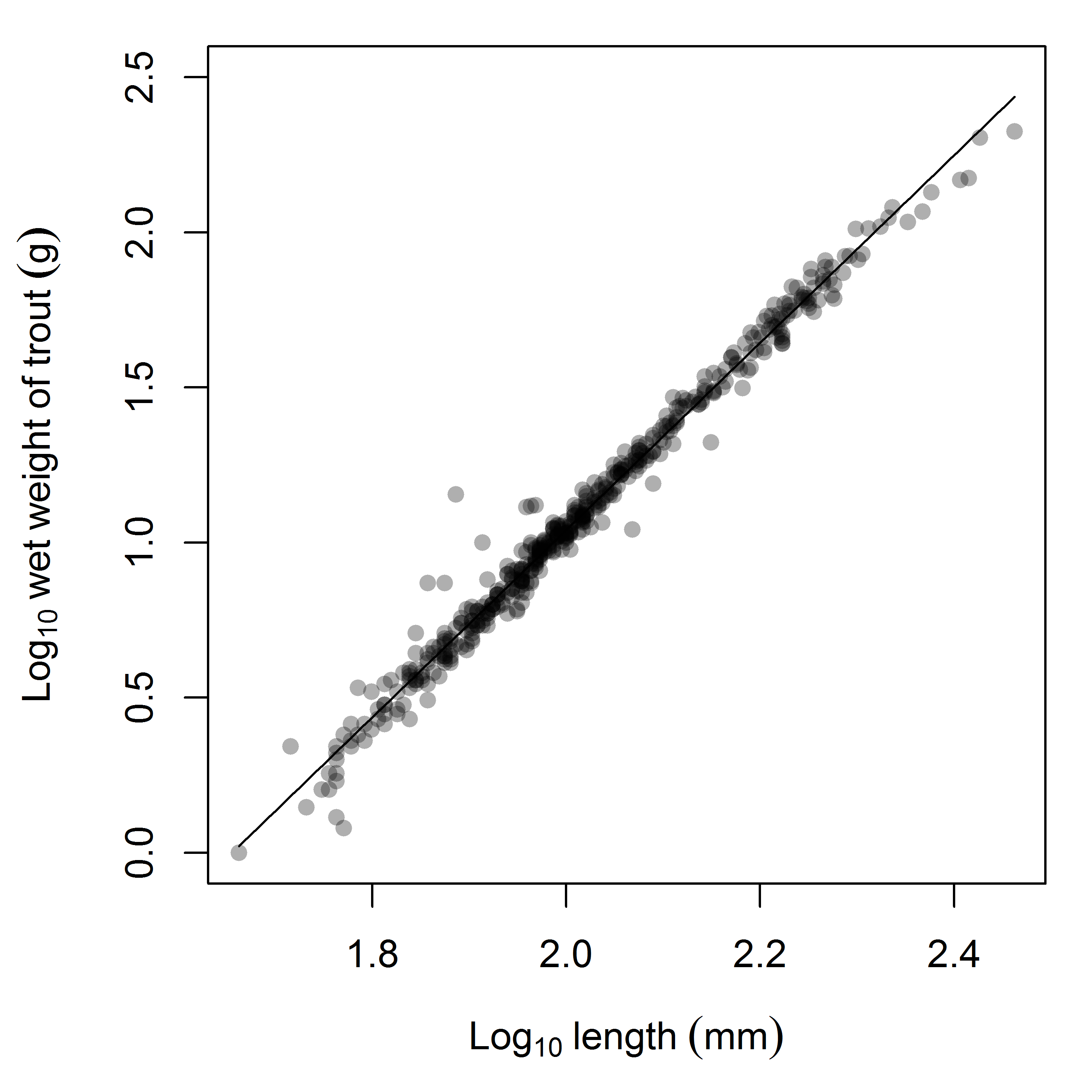


**Fig. S3.** Scale radius to fish length relationships used in the estimation of trout growth from scale circuli. Scale radius (mm) was measured as the distance from the scale centre to the last visible circulus; fish length corresponds to fork length (mm). Separate relationships were developed for six streams in the Hengill system from data collected in August 2004, October 2006, and August 2012. Linear regression IS1: *y* = 2.98*x* – 36.104, *F*_1,20_ = 38.15, *p* < 0.001, *r*^2^ = 0.64; IS5: *y* = 2.77*x* – 38.477, *F*_1,17_ = 108.71, *p* < 0.001, *r*^2^ = 0.86; IS6: *y* = 3.40*x* – 20.924, *F*_1,18_ = 85.21, *p* < 0.001, *r*^2^ = 0.82; IS8: *y* = 3.17*x* – 23.968, *F*_1,7_ = 89.27, *p* < 0.001, *r*^2^ = 0.92; IS12: *y* = 2.96*x* – 37.496, *F*_1,23_ = 114.13, *p* < 0.001, *r*^2^ = 0.82; IS14: *y* = 3.15*x* – 4.447, *F*_1,2_ = 720.47, *p* = 0.001, *r*^2^ = 1.00.


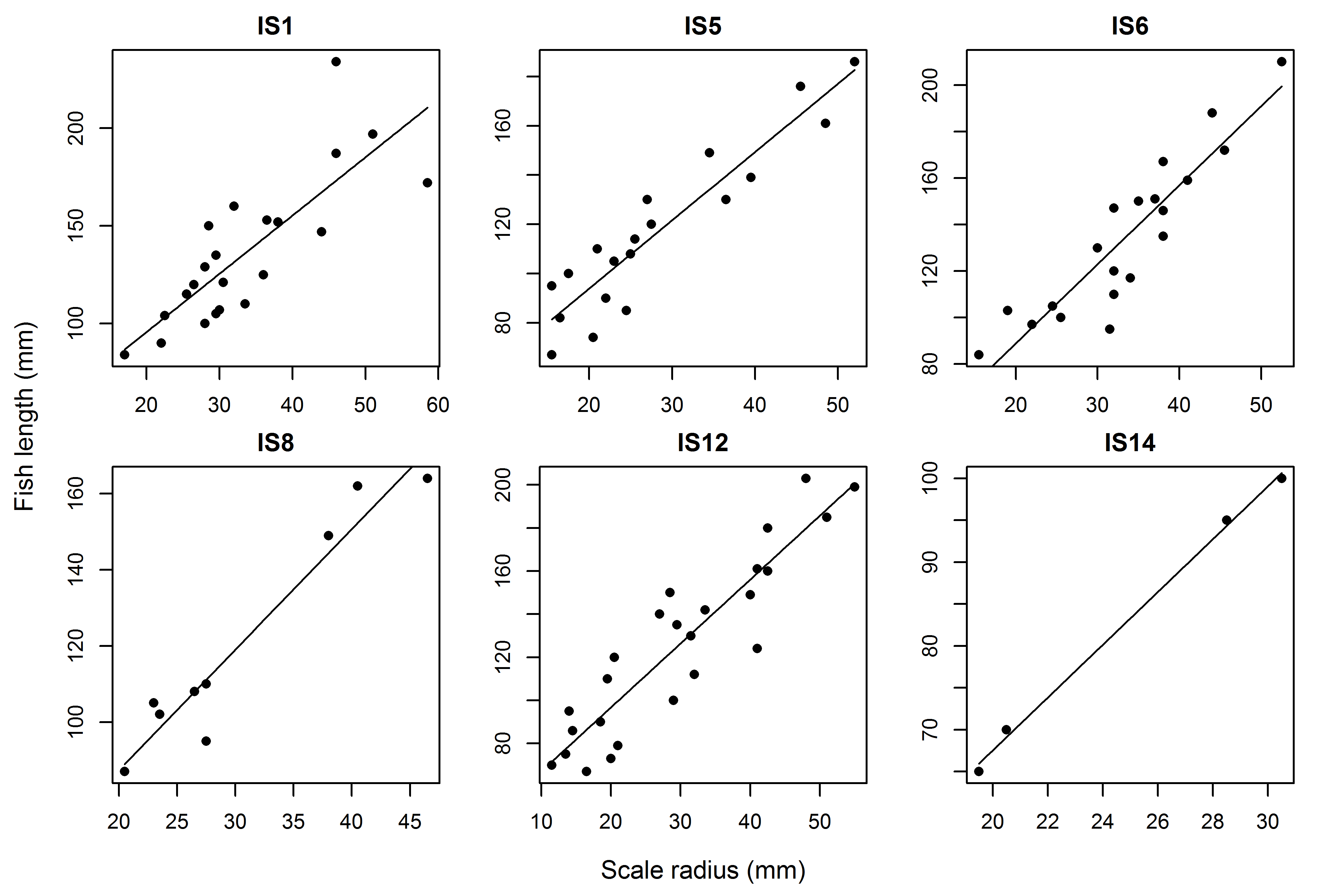


**Fig. S4.** Dietary niche width of trout and invertebrates in five streams in the Hengill system in 2004, 2006, and 2007. Cold streams (IS12 and 14) are shown in blue; warm streams (IS1, 5, and 8) are shown in red. We did not have sufficient data to calculate dietary niche width for all streams in all years (see Table S1). Note that the trout dietary niche is narrower in the warm streams, despite the fact their invertebrate prey tend to have a broader niche in the warmer environment.


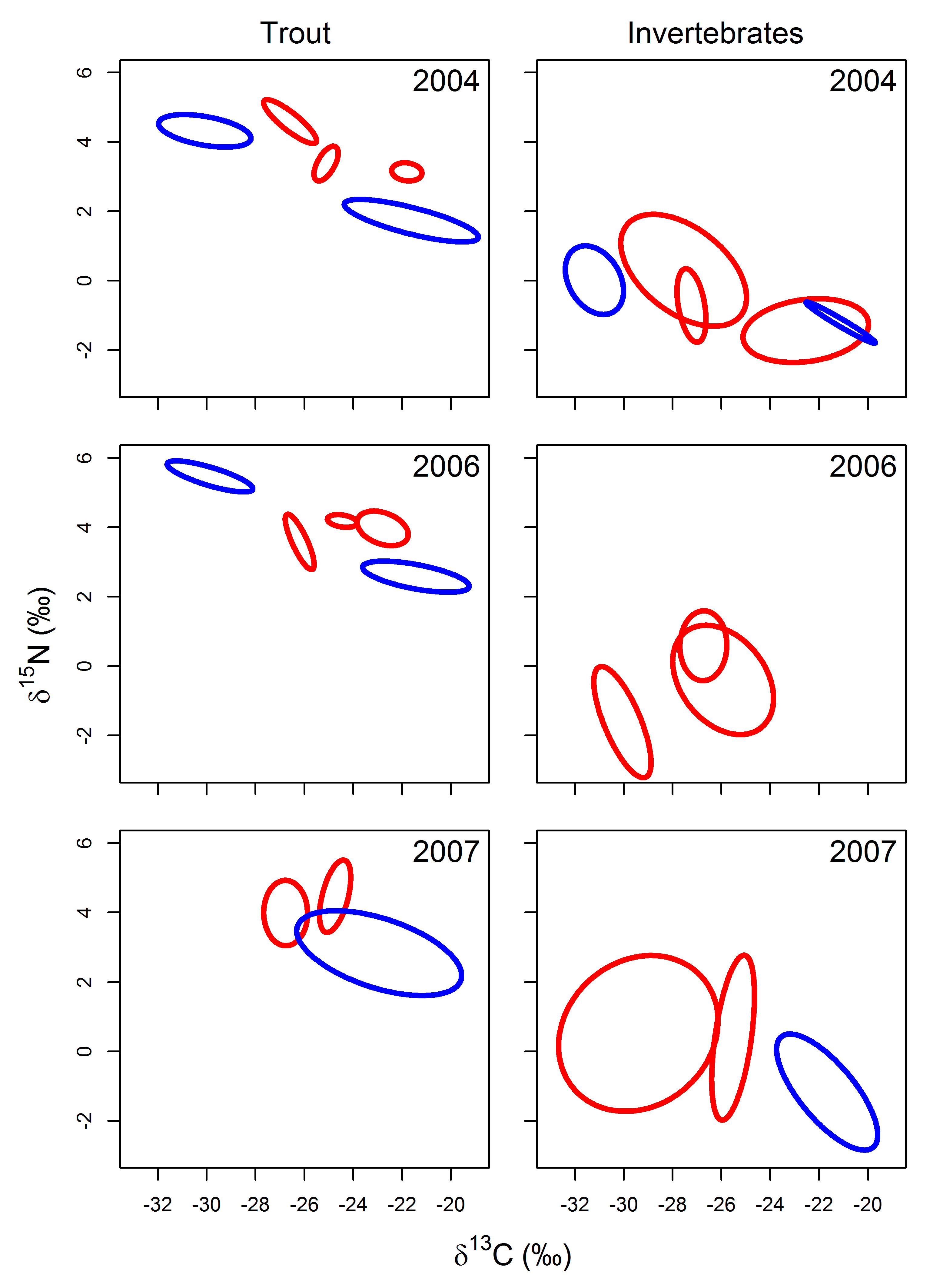


**Fig. S5.** Selectivity in the feeding of trout on common prey groups in August of 2004 (circles) and 2008 (crosses). Darker symbols indicate one or more points overlaying each other. Prey items include the freshwater snail (*Radix balthica*), blackfly larvae (Simuliidae), midge larvae (Chironomidae), predatory dipteran larvae, miscellaneous aquatic prey, and terrestrial subsidy. Solid black lines indicate the common linear regression across both years, while solid and dashed grey lines are the linear regressions for 2004 and 2008, respectively (if there was a significant main effect of year and/or interaction between year and temperature). See Table S3 for linear regression statistics. Note that trout selectivity is based on proportional data, so no units are displayed.


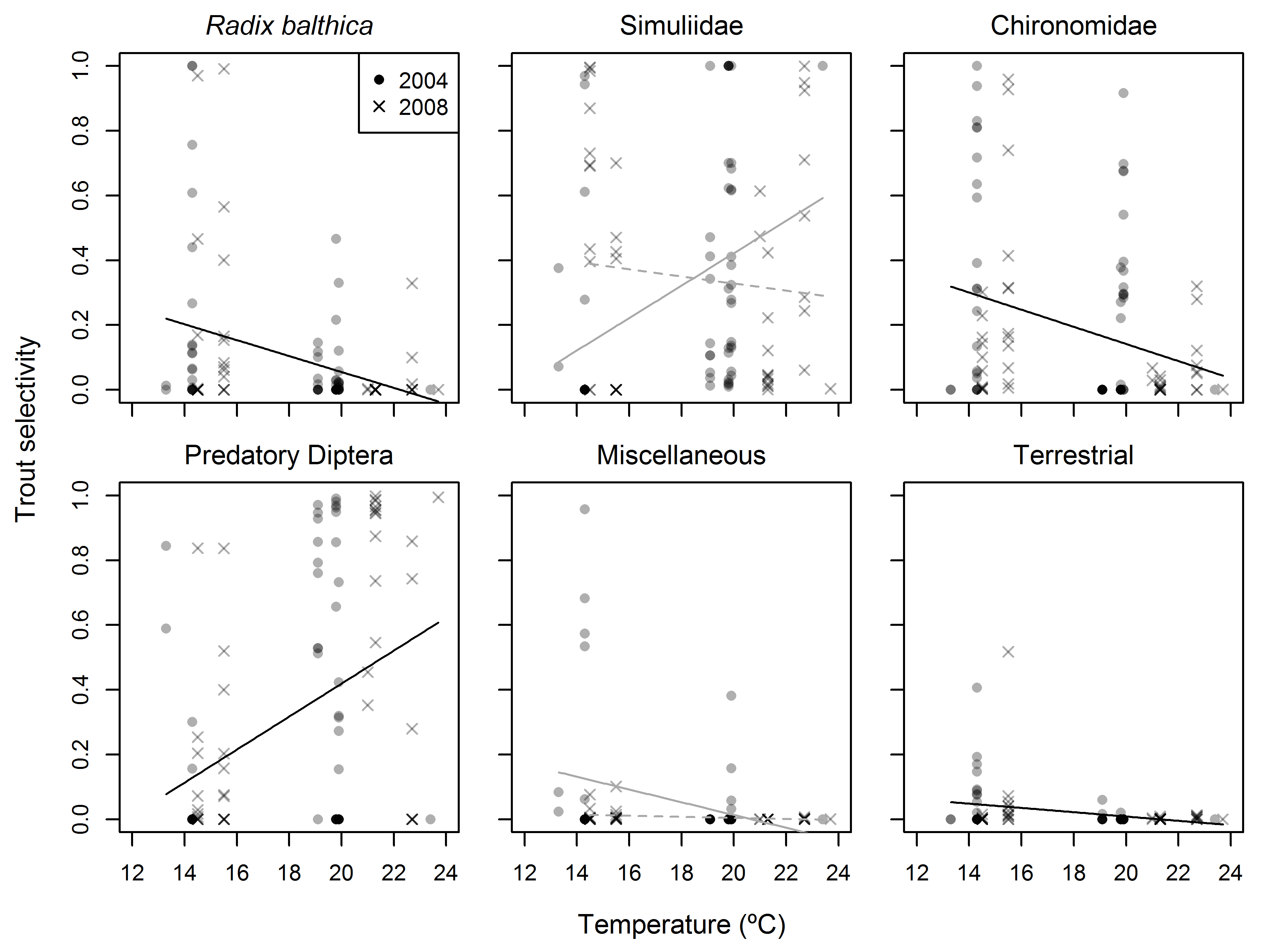

Supplement: Supplementary file 1 — Figure S1. Map of the Hengill geothermal valley. Figure S2. Length‐weight relationship for brown trout. Figure S3. Scale radius to fish length relationships. Figure S4. Dietary niche width of trout and invertebrates. Figure S5. Selectivity in the feeding of trout on common prey groups. Table S1. Sample sizes for estimating dietary niche width of trout and invertebrates Table S2. Details of sampling occasions during the trout mark‐recapture study Table S3. Linear regression statistics for selectivity of trout feeding [file GCB-22-3206-s001.doc]
